# Sulfonamides-induced oxidative stress in freshwater microalga *Chlorella vulgaris*: Evaluation of growth, photosynthesis, antioxidants, ultrastructure, and nucleic acids

**DOI:** 10.1038/s41598-020-65219-2

**Published:** 2020-05-19

**Authors:** Shan Chen, Liqing Wang, Wenbo Feng, Mingzhe Yuan, Jiayuan Li, Houtao Xu, Xiaoyan Zheng, Wei Zhang

**Affiliations:** 1 Centre for Research on Environmental Ecology and Fish Nutrient of the Ministry of Agriculture, Key Laboratory of Exploration and Utilization of Aquatic Genetic Resources, Ministry of Education, Shanghai Ocean University, Shanghai, 201306 China; 2 School of Agriculture and Biology, Shanghai Jiao Tong University, Shanghai, 200240 China; 3 Shanghai Aquatic Environmental Engineering Co., Ltd, Shanghai, 200090 China

**Keywords:** Freshwater ecology, Physiology

## Abstract

Sulfadiazine (SD), sulfamerazine (SM1), and sulfamethazine (SM2) are widely used and disorderly discharged into surface water, causing contamination of lakes and rivers. However, microalgae are regard as a potential resource to alleviate and degrade antibiotic pollution. The physiological changes of *Chlorella vulgaris* in the presence of three sulfonamides (SAs) with varying numbers of –CH_3_ groups and its SA-removal efficiency were investigated following a 7-day exposure experiment. Our results showed that the growth inhibitory effect of SD (7.9–22.6%), SM1 (7.2–45.9%), and SM2 (10.3–44%) resulted in increased proteins and decreased soluble sugars. Oxidative stress caused an increase in superoxide dismutase and glutathione reductase levels but decreased catalase level. The antioxidant responses were insufficient to cope-up with reactive oxygen species (hydrogen peroxide and superoxide anion) levels and prevent oxidative damage (malondialdehyde level). The ultrastructure and DNA of SA-treated algal cells were affected, as evident from the considerable changes in the cell wall, chloroplast, and mitochondrion, and DNA migration. *C. vulgaris*-mediated was able to remove up to 29% of SD, 16% of SM1, and 15% of SM2. Our results suggest that certain concentrations of specific antibiotics may induce algal growth, and algal-mediated biodegradation process can accelerate the removal of antibiotic contamination.

## Introduction

The increase in the use of antibiotics in human and veterinary medicine has raised concerns about their environmental accumulation^1^. The accumulation of sulfonamides (SAs), the antibacterial agents that inhibit dihydrofolic acid synthesis, is of particular interest^2^. Although the recorded environmental levels of SAs are usually within the microgram per litre range, this concentration may increase bacterial resistance and magnify toxicity via bioaccumulation in food chains^3^. Considering the non-degradability of SAs, modern sewage treatment plants may be insufficient to completely remove these agents^4^, resulting in their continuous presence in aquatic environment^5^. Thus, there is an urgent need to develop effective methods for SA-removal.

Freshwater algae form the most abundant biomass source in aquatic environments and are known to survive under extremely hostile environments^6,7^. Several studies have reported the ability of algae to effectively remove persistent organic pollutants such as pesticides^8^, phenols^9,10^, and estrogen^11,12^, and algae can uptake organic pollutants as carbon sources for their growth^7^. Thus, algae are considered as a potential resource to alleviate and degrade antibiotic pollution in surface water^13^. However, to the best of our knowledge, studies investigating the removal of SAs using algae are rare.

The understanding of the ability of freshwater green algae to tolerate and remove SAs may provide information on their potential application to curb antibiotic pollution. Here, we evaluate the oxidative damage to *Chlorella vulgaris* separately exposed to sulfadiazine (SD), sulfamerazine (SM1), and sulfamethazine (SM2) at various concentrations and determine its ability to remove these three SAs. *C. vulgaris* is one of the most common green algae found in freshwater systems. Furthermore, we discuss the association between the high number of –CH_3_ substituents in heterocyclic groups of SAs and their toxicity. This is the first study to determine the efficiency of a particular algal species to remove high concentrations of antibiotics with similar structures.

## Results

### Effects on algal growth

**Figure 1:**
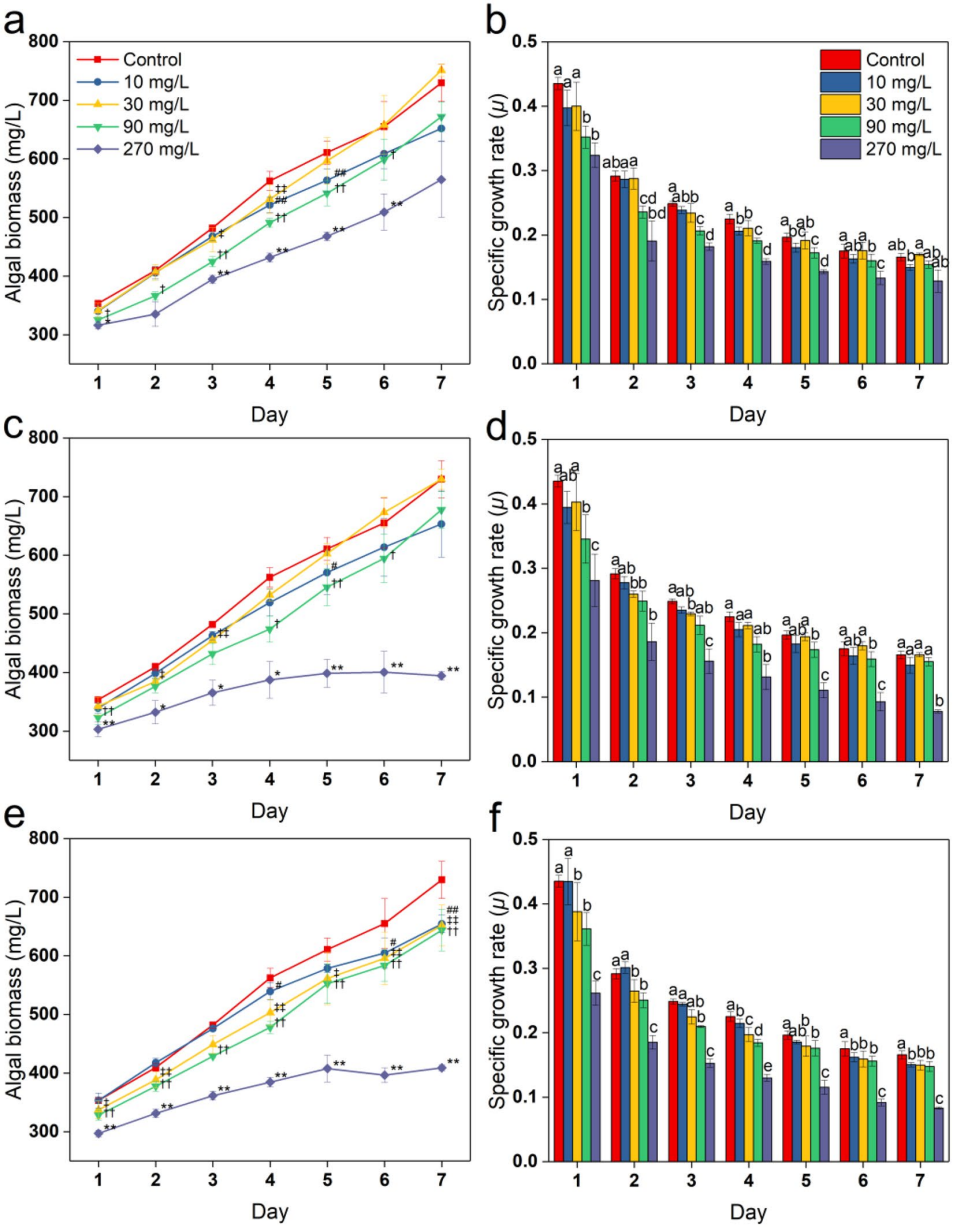
Effects of different concentrations of SAs on algal growth in terms of biomass and *μ*. Treatment with SD (**a**) and (**b**), SM1 (**c**) and (**d**), and SM2 (**e**) and (**f**). The symbol of ^‘#’, ‘‡’, ‘†’^ and ‘*’ represent significant difference between the BC group and L/D groups (10–270 mg/L), respectively. Error bars represent standard deviation (n = 4). Columns with different letters indicate significant differences (*p* < 0.05). ^#, ‡, †^ or *:*p* < 0.05; ^##, ‡‡, ††^ or **:*p* < 0.01.

The biomass of *C. vulgaris* was monitored every day during the study period under following conditions: L/D and BC. According to the experimental data, significant differences were observed in the algal biomass between BC group and L/D groups (*p* < 0.05), such that the biomass ranged from 353.29 to 729.61 mg/L in BC group (Fig. 1a,c,e). In comparison with the control group, *C. vulgaris* cultivated in the presence of SD, SM1, and SM2 showed a decrease in biomass by 7.9–22.6%, 7.2–45.9%, and 10.3–44%, respectively, except for the group treated with 30 mg/L SD and SM1. At day 7, the growth of the alga was significantly inhibited at 270 mg/L of SM1 and all concentrations of SM2. And the biomass tended to increase upon treatment with 30 and 90 mg/L of SD and SM1 comparing with control and 10 mg/L.

The values of *μ* for *C. vulgaris* cultivated under different SA-concentrations during the 7-day experimental period are shown in Fig. 1b,d,f. These values showed a decreasing trend with an increase in the concentration of SAs. At day 7, the value of *μ* slightly increased at 30 and 90 mg/L SD and SM1 as compared with that observed with 10 mg/L SD and SM1, and there was no significant difference only between the 270 mg/L SD and its control group. However, *μ* value decreased by 35.4–53%, and 36.5–49.9% in the groups treated with 270 mg/L of SM1 and SM2, respectively, during 7 days of incubation, in comparison with the *μ* value reported for the respective control group. The 96 h EC_10_ values of three SAs are shown in Table 1. The values of 96 h EC_10_ were in the order of SD > SM1 > SM2.

**Table 1:** The 96 h EC_10_ values of three SAs for *C. vulgaris*.

Antibiotics	Equation	R^2^	96 h EC_10_ (mg/L)
SD	y^a^ = 0.0867x^b^ + 6.1463	0.97	44.45
SM1	y = 0.1338x + 5.4771	0.98	33.80
SM2	y = 0.1361x + 5.7336	0.98	31.35

^a^ Specific growth rate inhibition (%).

^b^ SA-concentrations (mg/L).

### Effects on chlorophyll fluorescence

**Figure 2:**
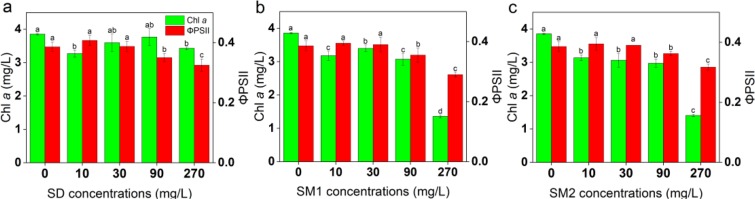
The chlorophyll *a* content and ΦPSII of *C. vulgaris* treated with SD (**a**), SM1 (**b**), and SM2 (**c**) after 7 days of incubation. Error bars represent standard deviation (n = 4). Columns with different letters indicate significant differences (*p* < 0.05) between the BC group and L/D groups.

The contents of chlorophyll *a* were significantly decreased for *C. vulgaris* treated with different concentrations of SAs except 30 and 90 mg/L SD than for the control cells at day 7 (Fig. 2). The highest chlorophyll *a* content in L/D groups was observed for *C. vulgaris* treated with 90, 30, and 10 mg/L of SD, SM1, and SM2, respectively, while the chlorophyll *a* content in the algae treated with 270 mg/L SD was higher than that detected in algae treated with 270 mg/mL of SM1 and SM2. The value of ΦPSII was found to significant decrease with an increase in SA-concentrations (90–270 mg/L). However, treatment with low concentrations (10–30 mg/L) of three SAs had no significant change.

### Effects on biochemical parameters

**Figure 3:**
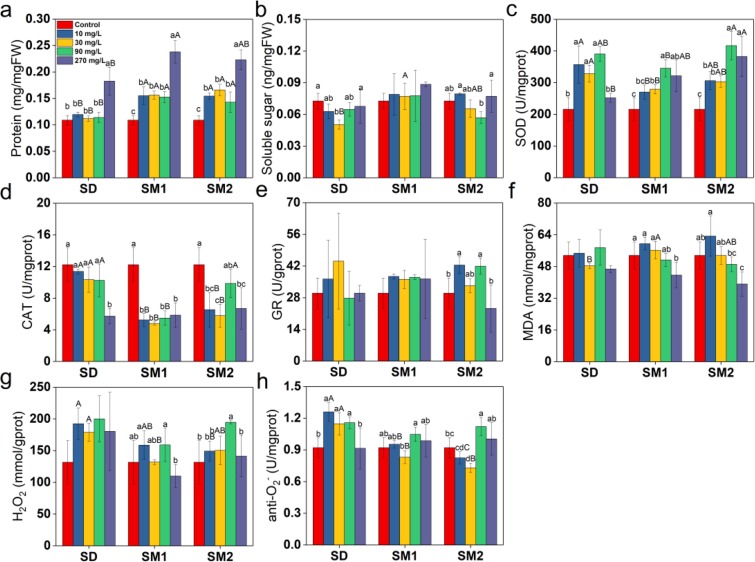
Biochemical parameters protein (**a**), soluble sugar (**b**), SOD (**c**), CAT (**d**), GR (**e**), MDA (**f**), H_2_O_2_ (**g**), and anti-·O_2_^−^ (**h**) of *C. vulgaris* at different concentrations of SAs after 7 days of incubation. Error bars represent standard deviation (n = 4). Uppercase letters represent differences between groups, and lowercase letters represent differences within groups (*p* < 0.05).

The protein content significantly increased following treatment with the four concentrations of SAs as compared with BC group (Fig. 3a). The highest protein contents reported after treatment with 270 mg/L of SD, SM1, and SM2 were 168%, 219% and 204% of the control, respectively. The soluble sugar content of all concentrations of SAs remained basically unchanged compared with BC group and significantly decreased in some SA-treatments, such as 30 mg/L SD and 90 mg/L SM2 (Fig. 3b).

With increasing SA-concentrations, the SOD activities increased, reaching the highest values at 10 mg/L SOD for SD, but at 90 mg/L SOD for SM1 and SM2 (Fig. 3c). The maximum activities of SOD were 181%, 160%, and 193% of the control, respectively. SOD activity reflected the toxic effects of 270 mg/L SAs in the order of SD < SM1 < SM2. However, CAT activity decreased following antibiotic treatment (Fig. 3d) and GR activity was most susceptible to SM2 of the three antibiotics (Fig. 3e).

MDA did not change significantly under the four different SD exposure levels and from 0 to 30 mg/L of SM1 and SM2 exposure levels, but with higher concentration of SM1 and SM2 it decreased (Fig. 3f). The content of H_2_O_2_ in *C. vulgaris* increased upon treatment with all concentrations of SAs (Fig. 3g). The anti-·O_2_^−^ activity was significantly increased in the algae treated with 10 and 30 mg/L of SD as compared with those treated with the same concentrations of SM1 and SM2 (Fig. 3h).

### Effects on ultrastructure

**Figure 4:**
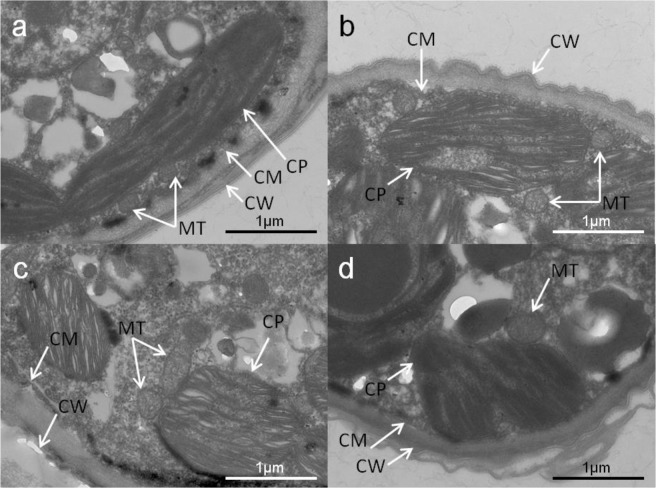
TEM images (×15,000) of *C. vulgaris* incubated for 7 days without and with 270 mg/L of different SAs: control (**a**); SD (**b**); SM1 (**c**); SM2 (**d**). CW: cell wall; CM: cell membrane; CP: chloroplast; MT: mitochondria.

The damage caused to the ultrastructure could be visualised with TEM for the cells from BC group and L/D groups cultivated in the presence of 270 mg/L SAs (Fig. 4). We observed more single cells in BC group than in L/D groups (see Supplementary Fig. S1). A typical cell from BC group showed a rigid cell wall, while the cell walls showed wrinkles and separated from cell membranes in the cells from L/D group. The lobed chloroplast showed a neat distribution and was located at the periphery. Thylakoids appeared mostly in three- or two-layered stacks throughout the chloroplast. In comparison, the chloroplasts appeared irregular and the thylakoids appeared disordered following L/D groups, suggestive of the SA-mediated inhibition of photosynthetic in the algae. We also observed significant changes in mitochondria. The mitochondrial size was larger in L/D groups than in BC group.

### Effects on nucleic acids

**Figure 5:**
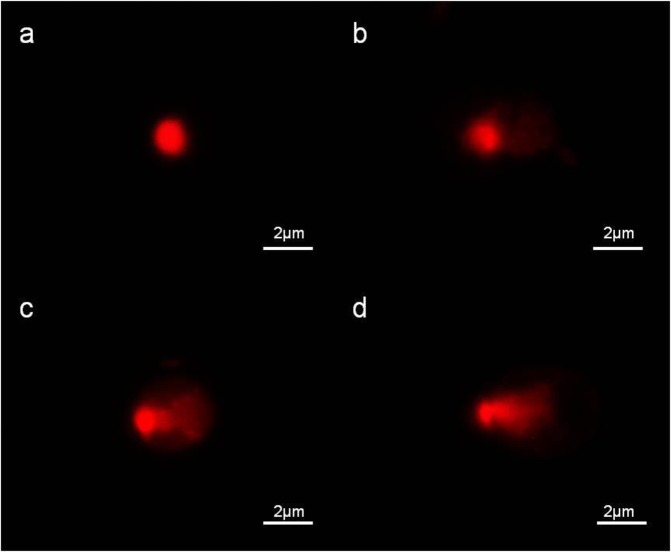
Effects on DNA integrity of *C. vulgaris* incubated for 7 days without and with 270 mg/L of different SAs: control (**a**); SD (**b**); SM1 (**c**); SM2 (**d**).

DNA damage observed in the cells from BC group and L/D groups containing 270 mg/L SAs are shown in Fig. 5. The cells from BC group had a mean tail DNA value of 2.1%. The mean values of tail DNA were 28.6%, 30.1%, and 32.5% in algae treated with SD, SM1, and SM2, respectively.

### Removal of antibiotics

**Figure 6:**
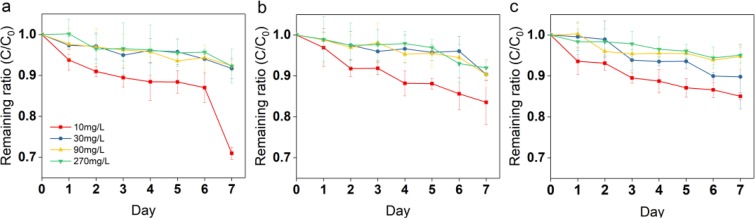
Removal of SD (**a**), SM1 (**b**) and SM2 (**c**) by *C. vulgaris* during 7 days of incubation under four concentrations. Error bars represent standard deviation (n = 4).

The measured exposure concentrations of SAs were within 106–117% of nominal concentrations. The degradation rates of SD, SM1, and SM2 for the algae from MC and DC groups via photolysis and hydrolysis were 6.5% and 1.7%, 4.9% and 1.2%, and 3.7% and 1.5%, respectively. *C. vulgaris*-mediated could remove 8%–29% of SD, 8%–16% of SM1, and 5%–15% of SM2 after 7 days of incubation (Fig. 6).

## Discussion

Cell biomass and *μ* value are the commonly used parameters to quantify algal growth^14,15^. These antibiotics at some concentrations range (i.e., 30 and 90 mg/L SD and SM1) may serve as carbon sources to promote algal growth, which is consistent with the previous reports^16,17^, wherein 5 and 1 mg/L levofloxacin improved the dry cell weight of *C. vulgaris* and *Scenedesmus obliquus*, respectively. Wang *et al*.^18^ found that the recovery capacity of algal species was consistent with the decrease in the growth inhibition effects during incubation, as observed following SD treatment in the present study. EC_10_ is a commonly used measurement endpoint to evaluate toxicity in ecological risk assessment and ecotoxicology^19^. These results are consistent with the reported for *C. vulgaris*, which showed low sensitivity to SAs^20^, although SD (72 h EC_50_: 2.19 mg/L) and SM1 (72 h EC_50_: 11.9 mg/L) for *Scenedesmus vacuolatus* were slightly more potent^21^. Taken together, the results of algal biomass, *μ*, and 96 h EC_10_ value used as the comprehensive factors to determine the phytotoxicity of pollutions to algae^22,23^ indicated that *C. vulgaris* was more sensitive to SM2, followed by SM1 and SD.

Chlorophyll *a* fluorescence is a sensitive parameter under environmental stress conditions, acting as an indicator of energy conversion in photosynthetic organisms, and it provides a relatively strong signal for Chlorophyll *a* concentration^24^, which has been used as a potential biomarker for the evaluation of antibiotic stress^25^. Chlorophyll biosynthesis is altered upon exposure to pharmaceuticals, resulting in the inhibition of algal growth^26^. In the present study, the decrease in chlorophyll content was consistent with algal growth inhibition (Figs. 1 and 2), which may be attributed to the reactive oxygen species (ROS)-mediated damage to the photosystem and chlorophyll biosynthesis in algae^27^. The relatively high chlorophyll content of cells may serve as a protective mechanism to scavenge the accumulated ROS in chloroplasts^28^. Thus, the scavenging efficiencies of *C. vulgaris* for ROS induced by SAs were in the order of SD > SM1 > SM2. The ΦPSII is considered as a main indicator for assessment of the photosystem II efficiency and for the photosynthetic capacity of algae^18^. In the present study, the value of ΦPSII was significantly inhibited at high doses (90–270 mg/L) at day 7, which may be ascribed to the disturbance of chlorophyll synthesis under stress of high levels of SAs as chlorophyll *a* is particularly vital for the functioning of PS II^29^.

The result of the contents of protein increased in response to the treatment of microalgae with antibiotics as compared with control microalgae was also observed by Aderemi *et al*.^30^. This observation may be attributed to the increase in enzyme synthesis or other energy-producing fractions and a corresponding increase in energy expenditure^30,31^. Soluble sugar plays an important role in carbon partitioning, photosynthesis, and osmotic homeostasis^32^. Any decrease in the soluble sugar content may be associated with reduced photosynthesis, inhibition of cell division, and osmotic imbalance^33^. SOD neutralises ·O_2_^−^ as a primary scavenger and catalyzes its conversion to H_2_O_2_, which is eventually scavenged by CAT to H_2_O^34,35^. The fact that SM2 induced SOD activity suggests that the rates of ROS scavenging and H_2_O_2_ regeneration were increased^36^. The effect was similar to that in other algae exposed to many other environmental contaminants such as herbicide^37^ and dibutyl phthalate^38^, and may be interpreted as a consequence of the oxidative stress associated with the overproduction of ROS^39^. On the other hand, SD induced no obvious changes in the enzyme activity. CAT activity is indicative of the ability of the alga to ameliorate toxicity under stress condition^40^. GR maintains the redox balance in the cellular environment by converting glutathione disulphide (GSSG) to glutathione (GSH) pool in the cytoplasm under stress conditions^41^. The decline in CAT and GR activities could be explained by they were activated unsuccessfully and changed digressively under various concentrations of SAs exposure, so that the alga is not fully competent to remove H_2_O_2_^42^, which is a non-radical ROS involved in signalling in normal cells and causes toxicity at high concentrations^43^. The increase in GR activity may result in rise in the capacity of quenching ROS^36^, as well as the increased anti-·O_2_^−^ activity presented in the present study, which mainly affected by non-enzymatic antioxidants which act to extinguish ROS and induce antioxidant enzyme activity^44^. As an ROS, ·O_2_^−^ induces significant oxidative damage through LPO and is harmful to organisms at high concentrations^45^. The increase of MDA indicates oxidative stress. The activity of MDA was negatively affected by SM1 and SM2. This may be related to the rise of SOD, attributed to its strongly effects on ROS scavenging.

Through TEM analysis, we found that SAs mainly affected cellular morphology, including cell wall, chloroplast, and mitochondrion. Mitochondrial amplification may be associated with the increase in energy requirement in response to adverse environmental conditions such as nutrient or toxicant stress^46^. Eguchi *et al*.^20^ reported that the inhibition of *C. vulgaris* growth in response to SAs was ameliorated following folic acid supplementation, indicating that the antibiotics inhibited folate synthesis in green algae. Thus, SAs may interfere with the normal cellular development and folic acid synthesis in the cytoplasm, mitochondrion, and chloroplast^47^.

Modifications in DNA integrity following exposure to organic pollutants may serve as early signals of potential degradation of ecosystems^48^. In the present study, the visible tail of comet and shrunken cell nucleus could be observed as per the study of Zhang *et al*.^49^, and indicated the moderate damage caused to algal cells. The increased nuclear fragmentation suggested that the toxic effects of SAs were in the order of SM2 > SM1 > SD. Białk-Bielińska *et al*.^21^ showed that the higher the number of –CH_3_ groups in the side R chain, the lower was the toxicity. This observation may be related to the difference in the pH of the medium environment and different p*K*a values, resulting in differences in bioavailability^21^.

Green algae may serve as mediators of antibiotic degradation in light conditions^50,51^. These results are in line with those previously reported, showing negligible contribution of abiotic factors in the removal of SAs^1^. The differences in the metabolism efficiencies of these three SAs are explained by Batista *et al*.^52^. The degradation of SM2 was slower than that of SM1 and SD because of the increase in the number of –CH_3_ and the corresponding increase in the steric hindrance to radical addition. The biodegradation, bioaccumulation, and/or bio-adsorption may play crucial roles in the removal of SAs.

## Materials and methods

### Microalgal culture

Axenic strain of *C. vulgaris* (Code: FACHB-8) originally purchased from the Freshwater Algae Culture Collection of the Institute of Hydrobiology (FACHB-Collection), Wuhan City, China, were cultivated in 500 mL Erlenmeyer flasks containing BG-11 media^53^. Cultures were maintained in a homoeothermic incubator at 25 °C ± 1 °C under 3000 lux illumination with a light-dark period of 12:12 h. To retain the exponential growth phase, algae were aseptically transferred to fresh media every 3–4 days^30^. Deionised water at 18.25 MΩ purity was obtained using Milli-Q Gradient-A 10 Millipore water deioniser.

### Experimental design

Stock solutions of the three test antibiotics, SD (CAS-No. 68–35–9), SM1 (CAS-No. 127–79–7), and SM2 (CAS-No. 257–68–1) (Table 2) purchased from Sigma-Aldrich (Steinheim, Germany) at> 98% purity were freshly prepared in BG-11 media before each toxicity test. Four concentrations (10, 30, 90, and 270 mg/L) of each antibiotic were used against *C. vulgaris*. The four test concentrations were attained by diluting a small amount of the stock solution in a geometric ratio (common ratio = 3) with the solutions of culture media for the microalgae. We used 0.1 mol/L sodium hydroxide (NaOH) as solvent to maintain pH 10 to fully dissolve SAs. Then, we added 0.1 mol/L hydrochloric acid (HCl) to adjust the initial pH 7 of the test medium^54^.

**Table 2:** Physicochemical properties of three SAs^71^.

Compound	Molecular formula	Molecular structure	Molecular weight	Log *K*ow	p*K*a
Sulfadiazine	C_10_H_10_N_4_O_2_S		250.28	−0.0314	6.52
Sulfamerazine	C_11_H_12_N_4_O_2_S		264.30	0.1944	7.10
Sulfamethazine	C_12_H_14_N_4_O_2_S		278.33	0.4687	7.40

Tests were carried out according to the OECD Test Guideline 201^55^ with minor modifications. To obtain sufficient biomass for the analysis of various parameters, the algal growth was performed in 250 mL Erlenmeyer flasks each containing 150 mL of test solution^3^. A specific volume of *C. vulgaris* culture in the exponential growth phase was diluted with a known volume of BG-11 medium autoclaved at 121 °C for 30 min to achieve a constant cell density (3.1 × 10^5^ cells/mL) in both the light/dark control (L/D; with antibiotics) and blank control (BC; without antibiotics) groups. Different concentrations of SAs and control were tested in four replicates (n = 4). In addition, dark control (DC; medium only spiked with 10 mg/L antibiotics and immediately wrapped with foil to prevent exposure to light) and medium control (MC; medium only spiked with 10 mg/L antibiotics under 12 h light/dark cycles) groups were evaluated in quadruplicates. The tests were carried out for 7 days under the same conditions used for the inoculums culture. For the uniform distribution of light, the positions of test flasks were randomised and changed every 24 h^56^.

### Measurement of cell growth and chlorophyll fluorescence

Samples of microalgal suspension (3 mL) were collected from 8:00 a.m. to 8:30 a.m. every day during the 7-day incubation^57^. Cell growth was determined at 679 nm wavelength using an ultraviolet-visible spectrophotometer (Unico UV-2800, Shanghai, China). The algal biomass was measured by algal volume based on a previously established methodology^58^. The value was converted to algal biomass (mg/L) based on the linear relationship between OD_679_ and biomass calculated as follows:

Cell biomass of *C. vulgaris* (mg/L) = 4021.9 × OD_679_ − 8.6817 (R² = 0.9995)

The specific growth rate (*μ*) was calculated by fitting the algal biomass to an exponential function using the following equation:

$$\mu =\frac{{\rm{l}}{\rm{n}}\,{N}_{2}-\,{\rm{l}}{\rm{n}}\,{N}_{0}}{{t}_{2}-{t}_{0}}$$

where *N*_2_ is the algal biomass at time *t*_2_ and *N*_0_ is the algal biomass at time *t*_0_.

The chlorophyll *a* fluorescence and effective quantum efficiency (ΦPSII) were measured with a pulse amplitude modulated fluorometer (Phyto-PAM Walz, Effeltrich, Germany) equipped with an emitter-detector-fibreoptic unit using an irradiance of 16 μmol photons·m^−2^·s^−1^ PAR and by an actinic light illuminating dark-acclimated cells at intensity equivalent to the incubation light (264 μmol photons·m^−2^·s^−1^ PAR), respectively^59^.

### Cell harvesting and enzyme extraction

Following incubation, algal cultures (10 mL) from BC group and L/D groups were harvested in 15 mL sterile tubes by centrifugation at 3,500 rpm for 10 min at 4 °C. The resultant pellet was resuspended in 0.01 mol/L sodium phosphate buffer (pH 7.4) and centrifuged. Homogenisation was carried out at 4 °C using a manual homogeniser and the enzymes obtained from the disrupted cells were extracted in 500 μL sodium phosphate buffer. The supernatant was collected for biochemical analysis.

### Analysis of energy substances

Assays were performed using traditional methods. Optical density values were measured with a spectrophotometer. The protein content in the crude extract was determined with the Coomassie Brilliant Blue (G-250) method, as reported by Bradford^60^ at 562 nm wavelength using bovine serum albumin as standard and expressed as milligram per milligram algal fresh weight (FW). Soluble sugar content was estimated as per the method described by Mccready *et al*.^61^ at 620 nm and expressed as nanogram per milligram FW.

### Analysis of antioxidant ability

Superoxide dismutase (SOD) activity was assayed by the nitro blue tetrazolium (NBT) method described by Beauchamp and Fridovich^62^. One unit (U) of SOD activity was defined as the amount of enzyme required to inhibit the rate of NBT reduction by 50% at 550 nm per milligram tissue protein. Catalase (CAT) activity was determined by measuring the initial rate of the decrease in the absorbance at 405 nm over 1 min at 30 °C in a spectrophotometric assay using hydrogen peroxide (H_2_O_2_)^63^. One U of CAT activity was defined as the degradation of 1 μmol H_2_O_2_ per second per milligram tissue protein. Glutathione reductase (GR) activity was determined at 340 nm with some modification in the microtiter plate assay described by Gutterer *et al*.^64^. One U of GR activity was defined as the degradation of 1 mmol nicotinamide adenine dinucleotide phosphate (NADPH) per minute per gram tissue protein.

### Analysis of oxidative damage

The level of malondialdehyde (MDA), a product of liquid peroxidation, was assessed with the evaluation of thiobarbituric acid reactive substance (TBARS). TBARS formed was measured with a spectrophotometer at 532 nm wavelength and quantified as MDA equivalent using 1,1,3,3-tetramethoxypropane as standard^65^. MDA content was expressed as nmol TBARS per milligram tissue protein. H_2_O_2_ level was estimated following the method described by Liu *et al*.^66^. H_2_O_2_ binds to molybdic acid to form a complex and the absorbance of H_2_O_2_ content was recorded at 405 nm wavelength and expressed as mmol per gram tissue protein. The ability of *C. vulgaris* to eliminate superoxide anions (·O_2_^−^), which are biologically produced by NADPH oxidase in metabolic processes to protect against invading pathogens, was determined using a commercially available inhibition and generation of ·O_2_^−^ kit (Nanjing Jiancheng Bioengineering Institute)^67^. One U of anti-·O_2_^−^ was defined as the decrease in the value relative to inhibition observed with 1 milligram vitamin C per gram tissue protein at 550 nm.

### Analysis of ultrastructure and comet assay

On day 7 of growth, two samples (10 mL) containing the highest concentrations of each antibiotic were centrifuged at 3,500 rpm for 10 min. The algal cells obtained from one sample were fixed with 2.5% glutaraldehyde and treated as per the modified method described by Lapaille *et al*.^68^ for microstructure analysis using transmission electron microscopy (TEM, HT7700, Hitachi, Japan).

The other sample was used for the detection of DNA damage with single cell gel electrophoresis or comet assay. The protocol used was as per the method described by Tice *et al*.^69^ with some modifications. Images were digitised using light and fluorescence microscopy with an Eclipse Ni epifluorescence microscope (Nikon) equipped with an inbuilt white and external mercury lamp light source (Model C-SHG1; Nikon). For documentation, a DS-Ri2 digital sight camera (Nikon) was used. Fifty nucleoids were evaluated from each group.

### Determination of antibiotic concentration

To determine the concentration of antibiotics in the test systems, the supernatants from samples were immediately decanted for further analysis during 7 days. Samples were filtered through 0.22 μm polytetrafluoroethylene (PTFE) filters prior to use for ultra-high performance liquid chromatography (UPLC) analysis. The instrument used was a ACQUITY UPLC H-Class system composed of a quaternary solvent manager, a sample manager FTN (Flow-Through Needle), a column heater and a photo-diode array (PDA). Empower 3 Pro software (version 2010) was used^70^. An ACQUITY UPLC BEH C18 chromatography column with dimensions 50 × 2.1 mm and particle size 1.7 µm was used for LC at a constant flow rate of 0.5 mL/min. A total of 2 μL of each sample was injected using an auto-sampler. The mobile phase comprised methanol and 1‰ formic acid in water (LC-MS grade; 10:90 v/v). The column oven temperature was controlled at 40 °C. Detection was performed at 256, 258 and 262 nm, respectively. Quantitative determination was performed using external standards. Limits of quantification (LOQ) and determination (LOD) were determined by using the respective calibration solutions, the LOQ of SD, SM1, and SM2 were 0.03, 0.05, and 0.05 mg/L, respectively, the LOD were 0.01, 0.02, and 0.02 mg/L, respectively.

### Statistical analysis

Percent growth inhibition was calculated for the response variable *μ*. The effective concentrations of SAs that inhibited algal growth by 10% (EC_10_) were calculated. Statistical differences in parameters between the BC group and L/D groups were analysed after normalisation with Shapiro-Wilk test, and homogeneity of variance was detected using Levene’s test by one-way analysis of variance (ANOVA) of SPSS statistics software (version 17, Chicago, IL, USA). A value of *p* < 0.05 was considered significant as per Fisher’s Least Significant Difference (LSD) and Tamhane post hoc tests. Comet analysis was carried out using CASP program (Casplab.com) to measure DNA migration parameters (i.e., % Tail DNA).

## Supplementary information

Supplementary Information.

## References

[1] Bai X, Acharya K (2016). Removal of trimethoprim, sulfamethoxazole, and triclosan by the green alga *Nannochloris* sp. J. Hazard. Mater..

[2] Isidori M, Lavorgna M, Nardelli A, Pascarella L, Parrella A (2005). Toxic and genotoxic evaluation of six antibiotics on non-target organisms. Sci. Total Environ..

[3] Xiong JQ, Kurade MB, Kim JR, Roh HS, Jeon BH (2017). Ciprofloxacin toxicity and its co-metabolic removal by a freshwater microalga *Chlamydomonas mexicana*. J. Hazard. Mater..

[4] Halling-Sørensen B (1998). Occurrence, fate and effects of pharmaceutical substances in the environment–a review. Chemosphere.

[5] Xin Y (2014). Toxicity evaluation of pharmaceutical wastewaters using the alga *Scenedesmus obliquus* and the bacterium *Vibrio fischeri*. J. Hazard. Mater..

[6] Coogan MA, Edziyie RE, Point TWL, Venables BJ (2007). Algal bioaccumulation of triclocarban, triclosan, and methyl-triclosan in a North Texas wastewater treatment plant receiving stream. Chemosphere.

[7] Xiong JQ (2016). Biodegradation of carbamazepine using freshwater microalgae *Chlamydomonas mexicana* and *Scenedesmus obliquus* and the determination of its metabolic fate. Bioresour. Technol..

[8] Gonzalez-Barreiro O, Rioboo C, Herrero C, Cid A (2006). Removal of triazine herbicides from freshwater systems using photosynthetic microorganisms. Environ. Pollut..

[9] Ji MK (2014). Biodegradation of bisphenol A by the freshwater microalgae *Chlamydomonas mexicana* and *Chlorella vulgaris*. Ecol. Eng..

[10] Zhou GJ, Peng FQ, Yang B, Ying GG (2013). Cellular responses and bioremoval of nonylphenol and octylphenol in the freshwater green microalga *Scenedesmus obliquus*. Ecotox. Environ. Safe..

[11] Hom-Diaz A (2015). Microalgae cultivation on wastewater digestate: β-estradiol and 17α-ethynylestradiol degradation and transformation products identification. J. Environ. Manage..

[12] Maes HM, Maletz SX, Ratte HT, Hollender J, Schaeffer A (2014). Uptake, elimination, and biotransformation of 17alpha-ethinylestradiol by the freshwater alga *Desmodesmus subspicatus*. Environ. Sci. Technol..

[13] Chen J, Zheng F, Guo R (2015). Algal feedback and removal efficiency in a sequencing batch reactor algae process (SBAR) to treat the antibiotic cefradine. PLoS One.

[14] Cheng J, Qiu H, Chang Z, Jiang Z, Yin W (2016). The effect of cadmium on the growth and antioxidant response for freshwater algae *Chlorella vulgaris*. SpringerPlus.

[15] Dauda S, Chia MA, Bako SP (2017). Toxicity of titanium dioxide nanoparticles to *Chlorella vulgaris* Beyerinck (Beijerinck) 1890 (Trebouxiophyceae, Chlorophyta) under changing nitrogen conditions. Aquat. Toxicol..

[16] Xiong JQ (2017). Biodegradation and metabolic fate of levofloxacin via a freshwater green alga, *Scenedesmus obliquus* in synthetic saline wastewater. Algal Res..

[17] Xiong JQ, Kurade MB, Jeon BH (2017). Biodegradation of levofloxacin by an acclimated freshwater microalga, *Chlorella vulgaris*. Chem. Eng. J..

[18] Misra, A. N., Misra, M. & Singh, R. Chlorophyll fluorescence in plant biology. (2012). Available at, http://www.intechopen.com/books/biophysics/chlorophyll-fluorescence-in-plant-biology

[19] Beasley A, Belanger SE, Brill JL, Otter RR (2015). Evaluation and comparison of the relationship between NOEC and EC_10_ or EC_20_ values in chronic *Daphnia* toxicity testing. Environ. Toxicol. Chem..

[20] Eguchi K (2004). Evaluation of antimicrobial agents for veterinary use in the ecotoxicity test using microalgae. Chemosphere.

[21] Białk-Bielińska A (2011). Ecotoxicity evaluation of selected sulfonamides. Chemosphere.

[22] Kurade MB, Kim JR, Govindwar SP, Jeon BH (2016). Insights into microalgae mediated biodegradation of diazinon by *Chlorella vulgaris*: microalgal tolerance to xenobiotic pollutants and metabolism. Algal Res..

[23] Zhao R (2017). Efficient enzymatic degradation used as pre-stage treatment for norfloxacin removal by activated sludge. Bioproc. Biosyst. Eng..

[24] Xiao Y, Huang Q, Chen L, Li P (2010). Growth and photosynthesis responses of *Phaeodactylum tricornutum* to dissolved organic matter from salt marsh plant and sediment. J. Environ. Sci..

[25] Perales-Vela HV, Garcia RV, Gomez-Juarez EA, Salcedo-Alvarez MO, Canizares-Villanueva RO (2016). Streptomycin affects the growth and photochemical activity of the alga *Chlorella vulgaris*. Ecotox. Environ. Safe..

[26] Nie X, Wang X, Chen J, Zitko V, An T (2010). Response of the freshwater alga *chlorella vulgaris* to trichloroisocyanuric acid and ciprofloxacin. Environ. Toxicol. Chem..

[27] Zhang L (2017). Salinity-induced cellular cross-talk in carbon partitioning reveals starch-to-lipid biosynthesis switching in low-starch freshwater algae. Bioresour. Technol..

[28] Kasahara M (2002). Chloroplast avoidance movement reduces photodamage in plants. Nature.

[29] Liu W, Ming Y, Huang Z, Li P (2012). Impacts of florfenicol on marine diatom *Skeletonema costatum* through photosynthesis inhibition and oxidative damages. Plant physiol. bioch..

[30] Aderemi AO (2018). Oxidative stress responses and cellular energy allocation changes in microalgae following exposure to widely used human antibiotics. Aquat. Toxicol..

[31] Sun X (2014). Effect of nitrogen-starvation, light intensity and iron on triacylglyceride/carbohydrate production and fatty acid profile of *Neochloris oleoabundans* HK-129 by a two-stage process. Bioresour. Technol..

[32] Rosa M (2009). Soluble sugars–metabolism, sensing and abiotic stress: a complex network in the life of plants. Plant Signal. Behav..

[33] Sami F, Yusuf M, Faizan M, Faraz A, Hayat S (2016). Role of sugars under abiotic stress. Plant Physiol. Biochem..

[34] Bigorgne E (2011). Ecotoxicological assessment of TiO_2_ byproducts on the earthworm *Eisenia fetida*. Environ. Pollut..

[35] Upadhyay AK (2016). Augmentation of arsenic enhances lipid yield and defense responses in alga *Nannochloropsis* sp. Bioresour. Technol..

[36] Nie XP, Liu BY, Yu HJ, Liu WQ, Yang YF (2013). Toxic effects of erythromycin, ciprofloxacin and sulfamethoxazole exposure to the antioxidant system in *Pseudokirchneriella subcapitata*. Environ. Pollut..

[37] Qian H (2009). The effect of exogenous nitric oxide on alleviating herbicide damage in *Chlorella vulgaris*. Aquat. Toxicol..

[38] Li FM (2015). Inhibitory effects and oxidative target site of dibutyl phthalate on *Karenia brevis*. Chemosphere.

[39] Marchi LD (2017). The impacts of seawater acidification on *Ruditapes philippinarum* sensitivity to carbon nanoparticles. Environ. Sci.: Nano..

[40] Rai UN, Singh NK, Upadhyay AK, Verma S (2013). Chromate tolerance and accumulation in *Chlorella vulgaris* L.: role of antioxidant enzymes and biochemical changes in detoxification of metals. Bioresour. Technol..

[41] Ding SH, Jiang R, Lu QT, Wen XG, Lu CM (2016). Glutathione reductase 2 maintains the function of photosystem II in *Arabidopsis* under excess light. Biochim. Biophys. Acta..

[42] Nemat Alla MM, Hassan NM (2006). Changes of antioxidants levels in two maize lines following atrazine treatments. Plant Physiol. Biochem..

[43] Singh R, Upadhyay AK, Singh DP (2017). Regulation of oxidative stress and mineral nutrient status by selenium in arsenic treated crop plant *Oryza sativa*. Ecotox. Environ. Safe..

[44] Foyer CH, Theodoulou LF, Delrot S (2001). The functions of inter- and intracellular glutathione transport systems in plants. Trends Plant Sci..

[45] Apel K, Hirt H (2004). Reactive oxygen species: metabolism, oxidative stress, and signal transduction. Annu. Rev. Plant Biol..

[46] Shao Y, Wu RSS, Kong RYC (2002). Physiological and cytological responses of the marine diatom *Skeletonema costatum* to 2,4-dichlorophenol. Aquat. Toxicol..

[47] Gambonnet B (2010). Folate distribution during higher plant development. J. Sci. Food Agric..

[48] Vasseur P, Cossu-Leguille C (2003). Biomarkers and community indices as complementary tools for environmental safety. Environ. Int..

[49] Zhang JW, Fu DF, Wu JL (2011). Synthesized oversulfated and acetylated derivatives of polysaccharide extracted from *Enteromorpha linza* and their potential antioxidant activity. Int. J. Biol. Macromol..

[50] Zhang JW, Ma L (2013). Photodegradation mechanism of sulfadiazine catalyzed by Fe(III), oxalate and algae under UV irradiation. Environ. Technol..

[51] Zhang JW, Fu DF, Wu JL (2012). Photodegradation of norfloxacin in aqueous solution containing algae. J. Environ. Sci..

[52] Batista APS, Pires FCC, Teixeira ACSC (2014). Photochemical degradation of sulfadiazine, sulfamerazine and sulfamethazine: relevance of concentration and heterocyclic aromatic groups to degradation kinetics. J. Photochem. Photobiol. A.

[53] Rippka R, Deruelles J, Waterbury JB, Herdman M, Stanier RY (1979). Generic assignments, strain histories and properties of pure cultures of cyanobacteria. J. Gen. Microbiol..

[54] Pan S, Yan N, Zhang Y, Rittmann BE (2015). UV photolysis for relieved inhibition of sulfadiazine (SD) to biomass growth. Bioproc. Biosyst. Eng..

[55] OECD Test No. 201: freshwater alga and cyanobacteria, growth inhibition test paris OECD Guidelines for the Testing of Chemicals, (2006).

[56] USEPA (United States Environmental Protection Agency) Short-term methods for estimating the chronic toxicity of effluents and receiving waters to fresh water organisms (EPA-821-R-02-013), 4th ed., USA Washington DC, (2002).

[57] Salbitani, G. *et al*. Sulfur Deprivation results in oxidative perturbation in *Chlorella sorokiniana* (211/8k). 56, 897-905 (2015). 10.1093/pcp/pcv015 25647328

[58] Hillebrand H, Dürselen C-D, Kirchtel D, Pollingher U, Zohary T (1999). Biovolume calculation for pelaglc and benthic microalgae. J. Phycol..

[59] Almeida AC, Gomes T, Langford K, Thomas KV, Tollefsen KE (2019). Oxidative stress potential of the herbicides bifenox and metribuzin in the microalgae *Chlamydomonas reinhardtii*. Aquat. Toxicol..

[60] Bradford MM (1976). A rapid method for the quantitation of microgram quantities of protein utilizing the principle of protein-dye binding. Anal. Biochem..

[61] Mccready RM, Guggolz J, Silviera V (1950). Determination of starch and amylose in vegetables. Anal. Biochem..

[62] Beauchamp C, Fridovich I (1971). Superoxide dismutase: improved assays and an assay applicable to acrylamide gels. Anal. Biochem..

[63] Góth L (1991). A simple method for determination of serum catalase activity and revision of reference range. Clini. Chim. Acta..

[64] Gutterer JM, Dringen R, Hirrlinger J, Hamprecht B (2010). Purification of glutathione reductase from bovine brain, generation of an antiserum, and immunocytochemical localization of the enzyme in neural cells. J. Neurochem..

[65] Ohkawa H, Ohishi N, Yagi K (1979). Assay for lipid peroxides in animal tissues by thiobarbituric acid reaction. Anal. Biochem..

[66] Liu L (2013). Oxidative stress induces gastric submucosal arteriolar dysfunction in the elderly. World. J. Gastroentero..

[67] Fang LC (2012). Characterization of *Rhodopseudomonas palustris* strain 2C as a potential probiotic. Apmis.

[68] Lapaille M (2010). Atypical subunit composition of the chlorophycean mitochondrial F_1_F_O_-ATP synthase and role of Asa7 protein in stability and oligomycin resistance of the enzyme. Mol. Biol. Evol..

[69] Tice RR, Andrews PW, Hirai O, Singh NP (1991). The single cell gel (SCG) assay: an electrophoretic technique for the detection of DNA damage in individual cells. Biol. React. Intermed. IV.

[70] D’Avolio A (2013). Ultra performance liquid chromatography PDA method for determination of tigecycline in human plasma. Ther. Drug. Monit..

[71] Chen S, Xu F, Zhang W, Tang WQ, Wang LQ (2019). Research progress in pollution situation and environmental behavior of sulfonamides. Environ. Chem..

