# Supplementary material for: Sulfonamides-induced oxidative stress in freshwater microalga Chlorella vulgaris: Evaluation of growth, photosynthesis, antioxidants, ultrastructure, and nucleic acids
Source: Sci Rep. 2020 May 19;10:8243. doi: 10.1038/s41598-020-65219-2 (PMC7237458; doi:10.1038/s41598-020-65219-2)
Supplement: Supplementary file 1 — Supplementary Information. [file 41598_2020_65219_MOESM1_ESM.pdf]

Sulfonamides-induced oxidative stress in freshwater microalga *Chlorella vulgaris*: Evaluation of growth, photosynthesis, antioxidants, ultrastructure, and nucleic acids

Shan Chen[1,*], Liqing Wang[1,*], Wenbo Feng[1], Mingzhe Yuan[1], Jiayuan Li[1], Houtao Xu[2], Xiaoyan Zheng[3] & Wei Zhang[1]

[1]Centre for Research on Environmental Ecology and Fish Nutrient of the Ministry of Agriculture, Key Laboratory of Exploration and Utilization of Aquatic Genetic Resources, Ministry of Education, Shanghai Ocean University, Shanghai 201306, China.

[2]School of Agriculture and Biology, Shanghai Jiao Tong University, Shanghai 200240, China.

[3]Shanghai Aquatic Environmental Engineering Co., Ltd, Shanghai 200090, China.

[*]These authors contributed equally to this work.

Correspondence and requests for materials should be addressed to W.Z. (email: weizhang@shou.edu.cn)

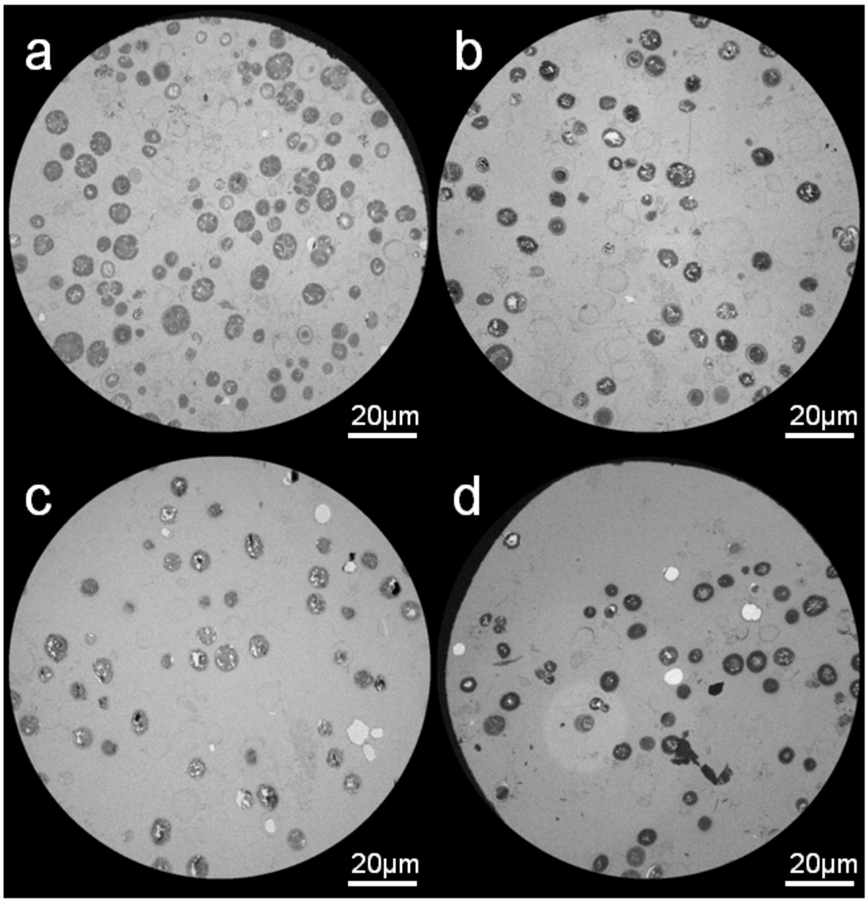

Supplementary Figure S1. TEM images (×12,000) of *C. vulgaris* cells incubated for 7 days without and with 270 mg/L of different sulfonamides: control (a); SD (b); SM1 (c); SM2 (d).
